# Proximal spleno-renal shunt with retro-aortic left renal vein in a patient with extra-hepatic portal vein obstruction: first case report

**DOI:** 10.1186/s12893-017-0262-6

**Published:** 2017-06-02

**Authors:** Sundeep Jain, Mukesh Kalla, Adil Suleman, Alok Verma

**Affiliations:** 1 Department of Gastrointestinal & HPB Surgery, Fortis Hospital, A-57, Apartment 203, Pearl Grands, Shanti Path, Tilak Nagar, Jaipur, 302004 India; 2 Department of Gastroenterology & Hepatology, S. R. Kalla Hospital, Jaipur, India; 3 Department of Anaesthesia, S. R. Kalla Hospital, Jaipur, India

**Keywords:** Portal hypertension, Spleno-renal shunt, Renal venous hypertension, Retro-aortic left renal vein

## Abstract

**Background:**

Presence of retro-aortic left renal vein poses special challenge in creating spleno-renal shunt potentially increasing the chance of shunt failure**.** The technical feasibility and successful outcome of splenectomy with proximal spleno-renal shunt (PSRS) with retro-aortic left renal vein is presented for the first time. The patient was treated for portal hypertension and hypersplenism due to idiopathic extra-hepatic portal vein obstruction.

**Case presentation:**

A twenty year old male suffering from idiopathic extra-hepatic portal vein obstruction presented with bleeding esophageal varices, portal hypertensive gastropathy, asymptomatic portal biliopathy and symptomatic hypersplenism. As variceal bleeding did not respond to endoscopic and medical treatment**,** surgical portal decompression was planned. On preoperative contrast enhanced computed tomography retro-aortic left renal vein was detected. Splenectomy with proximal splenorenal shunt with retro-aortic left renal vein was successfully performed by using specific technical steps including adequate mobilisation of retro-aortic left renal vein and per-operative pressure studies. Perioperative course was uneventful and patient is doing well after 3 years of follow up.

**Conclusions:**

PSRS is feasible, safe and effective procedure when done with retro-aortic left renal vein for the treatment of portal hypertension related to extra-hepatic portal vein obstruction provided that attention is given to key technical considerations including pressure studies necessary to ensure effective shunt. Present case provides the first evidence that retro-aortic left renal vein can withstand the extra volume of blood flow through the proximal shunt with effective portal decompression so as to treat all the components of extra-hepatic portal vein obstruction without causing renal venous hypertension.

## Background

Portal hypertension is related to extrahepatic portal vein obstruction (EHPVO) in the absence of cirrhosis of liver. In developing countries EHPVO is seen in one third of patients with variceal bleed [1]. Proximal splenorenal shunt (PSRS) with splenectomy and end to side splenorenal venous anastomosis is shown to be an effective and safe procedure for the treament of all the components of EHPVO viz. variceal bleeding, gastropathy, symptomatic hypersplenism and portal biliopathy [2, 3].

Left renal vein usually lies anterior to the aorta and in 1.8 – 3.4% cases there is a single left renal vein (LRV) in retro-aortic position before it enters inferior vena cava (IVC) [4]. The normal LRV has a greater length and is more superficial in position in comparison to retro-aortic LRV. Presence of retro-aortic LRV poses special challenge in creating spleno-renal shunt [5, 6] potentially increasing the chance of shunt failure as it restricts the ability to mobilize LRV which is essential for creating successful shunt. We couldnot find any evidence in the literature showing that retro-aortic LRV can withstand the extra volume of portal blood flow through the proximal shunt without causing renal venous hypertension and thus providing the effective portal decompression so as to treat all the components of EHPVO.

We have not encountered a single case of retro-aortic left renal vein in 33 cases of EHPVO that we have operated upon in last 9 years. Present case report describes important technical issues to ensure creation of effective PSRS with retroaortic LRV for the treatment of EHPVO related variceal bleed, gastropathy and symptomatic hypersplenism without causing renal venous hypertension.

## Case presentation

A twenty year old male, known case of EHPVO with portal hypertension and eradicated esophageal varices by endoscopic variceal ligation (EVL), presented to the emergency department with hematemesis and shock in June 2013. All the events in the course of his management are presented with timeline in Table 1.

**Table 1:** Timeline Table

Dates	Relevant past medical history (symptoms, diagnoses, interventions)		
At presentation on 05/06/2013	Growing lump left upper abdomen since last 15 years Recurrent variceal bleed since last 7 years Epistaxis and ecchymosis since last 3–4 years Diagnosed with – Portal hypertension due to Extrahepatic portal vein obstruction (EHPVO) Earlier managed with - Blood transfusions & EVL		
Dates	Summaries from initial & followup visits	Diagnostic testing (with dates)	Interventions
05/06/2013	Presented in emergency with hemetemesis & shock On examination- splenomegaly, no sign of liver failure Investigations- UGI endoscopy, Dupplex ultrasound, contrast enhanced computed tomogram, hemogram, liver function tests, coagulation tests, bone marrow examination renal function tests and urine examination Diagnosis- idiopathic EHPVO with acute variceal bleed, portal hypertensive gastropathy, symptomatic hypersplenism & evolving portal biliopathy	Hemogram (05/06/2013)- Hemoglobin 7.1 g/dl, total leucocyte count 1600/cumm, platelet count 45,000/cumm Liver function tests (05/06/2013)- normal Prothrombin time (05/06/2013)- normal Renal function tests (05/06/2013)- normal UGI endoscopy (06/06/2013)- four columns of grade II/III esophageal varices with post EVL ulcers, red colour sign & portal hypertensive gastropathy Protein C, S & antithrombin III levels (06/06/2013)- normal Bone marrow aspiration (06/06/2013)- normal cellularity with no abnormal cells, decreased myeloid erythroid ration (2:1) with normoblastic reaction Urine examination (07/06/2013)- normal Contrast enhanced computed tomogram (07/06/2013)- normal liver, portal vein replaced with large collaterals (portal cavernoma), 23 cm long splenomegaly with 12 mm calibre splenic vein at hilum. Multiple perisplenic, peri gallbladder & peri pancreatic collaterals seen with mild proximal biliary radical dilatation. Left renal vein was retro-aortic in position	Blood transfusions- 05/06/2013 to 07/06/2013- 6 units EVL- 05/06/2013, massive hemetemesis after 24 h Sengstaken-Blackmore tube- 06/06/2013 Condition stabilised Operation- Splenectomy with proximal end to side spleno-renal shunt done on 08/06/2013
08/06/2013 to 16/06/2013	Immediate postoperative outcome- Hemodynamically stable Sengstaken- Blackmore tube removed on 09/06/2013 No rebleed, encephalopathy, ascites	Liver biopsy (08/06/2013)- normal Hemogram (10/06/2013)- haemoglobin 9.3 g/dl, total leucocyte count 10,800/cumm, platelet count 2.1 lakh/cumm Liver function tests (10/06/2013)- normal Urine examination (10/06/2013)- normal UGI endoscopy (14/06/2013)- grade I residual varix, small post EVL ulcers with absence of red color sign & gastropathy	Vaccinated against Streptococcus pneumoniae, Hemophilus influenzae type B & Neisseria meningitides organisms on 16/06/2013 Discharged from hospital on 16/06/2013 Advised for early Physician consultation in case of any infective episode like respiratory infections
16/10/2013	History & Clinical examination- Normal No rebleed, encephalopathy, ascites & evidence of hypersplenism	UGI endoscopy (16/10/2013)- normal with no varices Contrast enhanced computed tomogram (16/10/2013)- patent spleno-renal shunt, fewer collaterals around gallbladder and hepatoduodenal ligament with decreased dilatation of intrahepatic biliary radicals. Hemogram (16/10/2013)- normal Liver function tests (16/10/2013)- normal Renal function tests- (16/10/2013)- normal Urine examination (16/10/2013)- normal	Advised for- Normal diet Early Physician consultation in case of any infective episode like respiratory infections
10/07/2016	History & Clinical examination- Normal No rebleed, encephalopathy, ascites & evidence of hypersplenism	UGI endoscopy (10/07/2016)- normal Duplex ultrasound (10/07/2016)- patent shunt Hemogram (10/07/2016)- normal Liver function tests (10/07/2016)-normal Renal function tests (10/07/2016)- normal Urine examination (10/07/2016)- normal	Normal diet Advised for early Physician consultation in case of any infective episode like respiratory infections Yearly follow up

First episode of hematemesis and malena occurred at the age of thirteen year and was managed elsewhere with blood transfusions and EVL for oesophageal variceal bleeding. Oesophageal varices were eradicated in 4 sessions of EVL. Subsequently the patient suffered another episode of malena one year before his presentation with us in 2013.

There was a history of growing lump in left upper abdomen for the last fifteen years. He also complained of epistaxis and ecchymosis for last 3–4 years, with pain while lying on left side of the abdomen. He never had jaundice, ascites, encephalopathy or pedal oedema. There was no history of omphalitis, umbilical vein catheterization and intra-abdominal sepsis after his birth and in early childhood. His family, personal and social histories were insignificant. His general physical examination was normal. On abdominal examination there was 12 cm long smooth and tender splenomegaly. The liver span was normal and there was no sign of encephalopathy, ascites and pedal oedema.

**Fig. 1:**
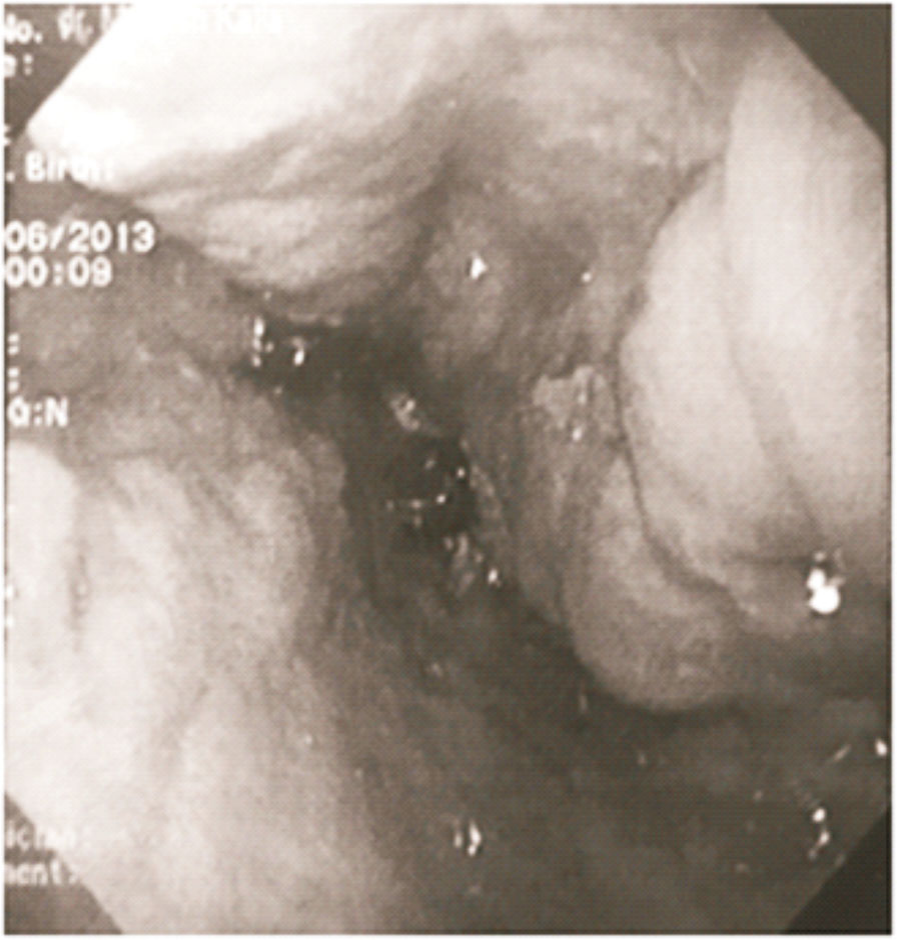
Pre-shunt UGI endoscopic findings showing grade III oesophageal varices

The upper gastrointestinal (UGI) endoscopy revealed 4 columns of grade II/III esophageal varices (Fig. 1) with post EVL ulcers, red colour sign along with fresh blood in esophagus and stomach and portal hypertensive gastropathy.

Duplex ultrasound of the abdomen showed a normal liver with portal vein replaced by large periportal collaterals (portal cavernoma), 23 cm long splenomegaly in cranio-caudal axis with 12 mm calibre splenic vein at the hilum. There were multiple perisplenic, peri-gallbladder and peripancreatic collaterals. The left renal vein was patent.

**Fig. 2:**
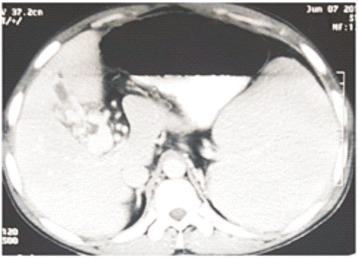
Multiple collaterals around gallbladder and hepatoduodenal ligament with dilated intrahepatic biliary radicals (portal biliopathy) with normal liver and no ascites

**Fig. 3:**
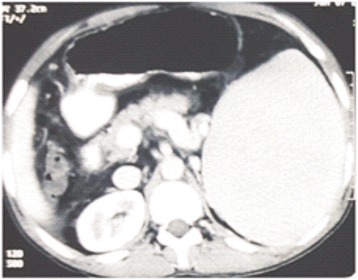
Splenomegaly with dilated splenic vein

**Fig. 4:**
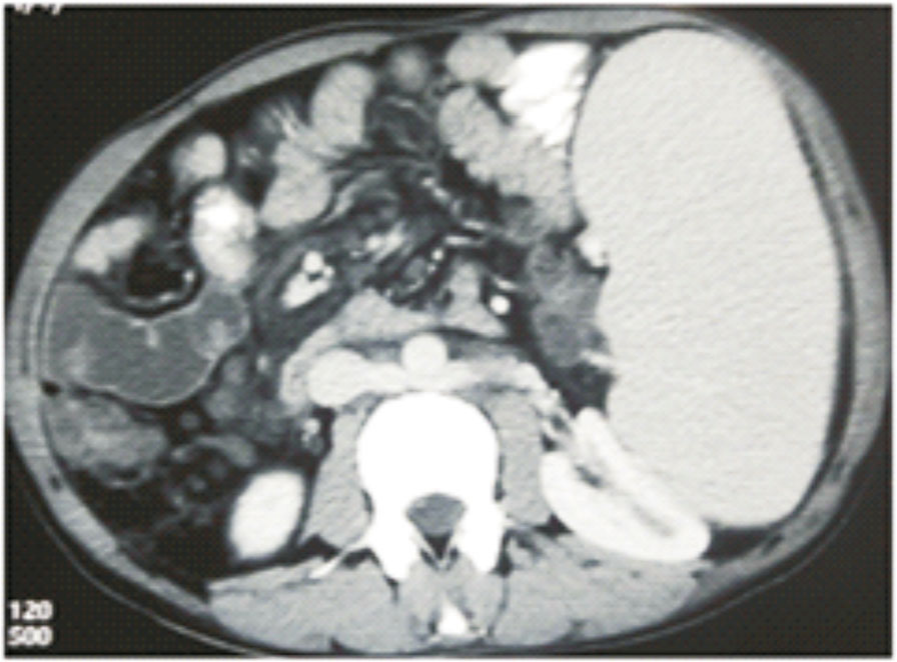
Retro-aortic LRV with enlarged spleen compressing *left* kidney

Contrast enhanced computed tomography confirmed the findings of Doppler scan and additionally revealed extra hepatic biliary obstruction due to periportal collaterals causing mild proximal biliary radical dilatation (Fig. 2), compressed left kidney due to enlarged spleen along with the presence of retro-aortic left renal vein (Figs. 3 and 4).

Hemogram showed a low haemoglobin of 7.1 g/dl with features of hypersplenism (low total leucocyte count 1600/cumm and platelet count 45,000/cumm).

Liver function tests were within normal limits with serum bilirubin 1.0 mg/dl, serum alkaline phosphatase 91 U/L, serum aspartate transaminase 28 U/L, serum alanine transaminase 22 U/L, serum albumin 3.4 g/dl with albumin globulin ratio of 1:1, normal prothrombin time (PT/INR- 15 s/ 1.3) and normal activated partial thromboplastin time (APTT) of 34 s.

Bone marrow aspiration was performed to rule out any other cause of low cellular count. It showed normal cellularity, absence of any abnormal cells, decreased myeloid erythroid ratio (2:1), with normoblastic reaction.

Protein C, protein S and antithrombin III levels were normal. Renal function tests and urine examination were also normal with no evidence of renal venous hypertension.

In view of all the findings the final diagnosis was EHPVO (idiopathic) with acute variceal bleeding, portal hypertensive gastropathy, symptomatic hypersplenism, and asymptomatic portal biliopathy.

Initially he was managed with blood transfusions (6 units), EVL and terlipressin infusion. He had massive hematemesis after 24 h of EVL which was controlled by Sengstaken-Blackmore tube**,** subsequently with the plan of splenectomy with PSRS once his condition stabilises. The options of mesocaval shunt and esophagogastric devascularisation were also considered in case the attempt at PSRS fails in view of retroaortic LRV.

The patient was given piperacillin- tazobactum and after 3 days of admission taken for splenectomy with proximal end to side spleno-renal shunt (PSRS) with the retroaortic LRV. Patient was placed in supine position with around 20 degree elevation of left flank by inserting a towel roll. A long left subcostal incision was made extending into the left flank and across the midline medially. Procedure was started by opening the lesser sac and ligating the splenic artery in continuity so as to allow spleen to shrink in size. The splenic vein was then dissected at the splenic hilum before splenectomy, so as to preserve maximum length of vein for tension free slenorenal shunt creation. Following splenectomy the segment of splenic vein on the posterior aspect of the pancreas was dissected. All the small pancreatic branches draining into splenic vein were dissected, ligated and divided individually so as to achieve adequate length of splenic vein for shunt formation. LRV was carefully dissected all around in its whole length from the left border of aorta to the renal hilum keeping in mind its deeper position and short length due to its retro-aortic position. Its mobility was further increased by ligating and dividing the left adrenal and gonadal veins.

An end to side tension free anastomosis was done between the end of splenic vein and the anterior wall of the LRV with continous 6–0 prolene with a growth factor. Heparin was not used. Pressures in the splenic vein, LRV and IVC were measured. The absolute basal portal pressure at the beginning of the operation and portal pressure after performing the shunt was 17 mmHg and 5 mmHg respectively. There was no pressure gradient across splenorenal venous anastomosis. The pressure gradient between LRV and IVC before the creation of shunt was 2 mmHg and post shunt gradient between the LRV and IVC was 6 mmHg. This ensured functional shunt and absence of renal venous hypertension. Trucut liver biopsy was taken and abdomen was closed without drains.

The operative time was 4 ½ hours with intraoperative blood loss of 150 ml. Patient remained hemodynamically stable throughout the operation and Sengstaken-Blackmore tube was removed 24 h later.

The postoperative course was uneventful with no rebleed, encephalopathy and ascites. Liver biopsy was normal with normal hemogram (hemoglobin- 9.3 g/dl, total leucocyte count- 10,800/cumm, platelet count – 2.1 lakh/cumm) and normal renal and liver function tests including prothrombin time and activated partial thromboplastin time. The UGIE on 6th postoperative day revealed grade I residual varix, small post EVL ulcers with absence of red colour sign and portal hypertensive gastropathy. Examination of urine remained normal in the postoperative period. He was discharged on 8th postoperative day after receiving vaccination against Streptococcus pneumoniae, Haemophilus influenzae type B and Neisseria meningitides organisms.

**Fig. 5:**
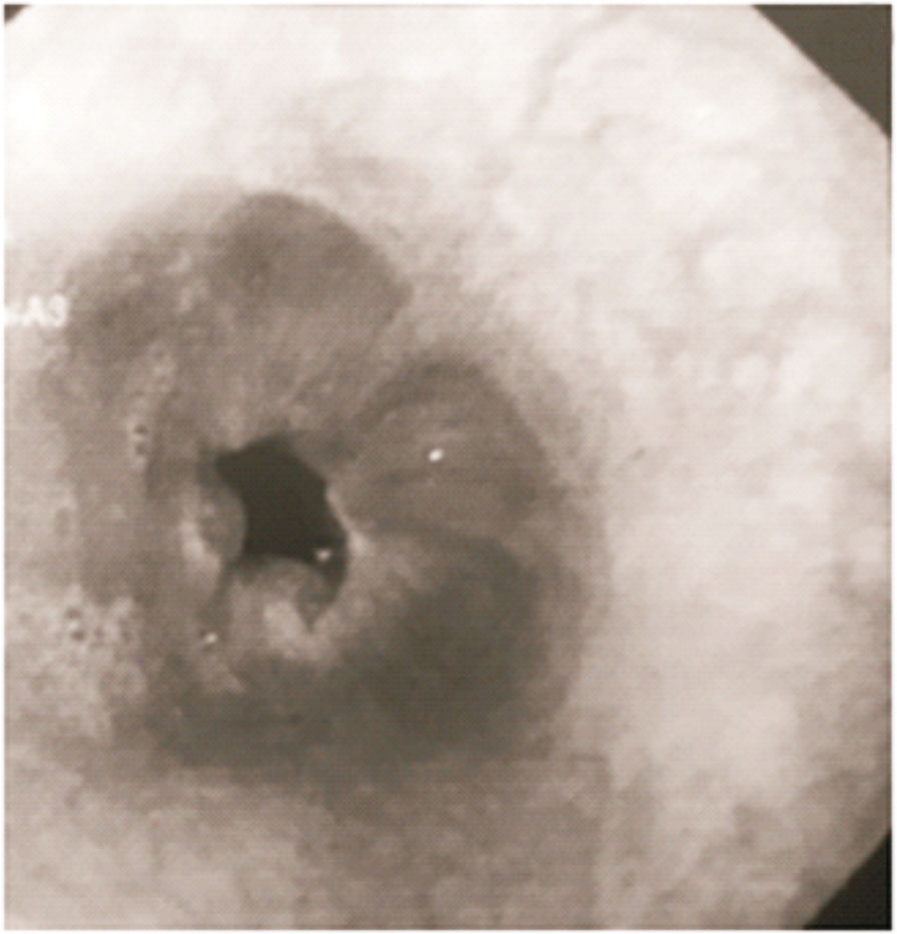
Post-shunt UGI endoscopic findings showing absence of varices after 4 months of shunt

**Fig. 6:**
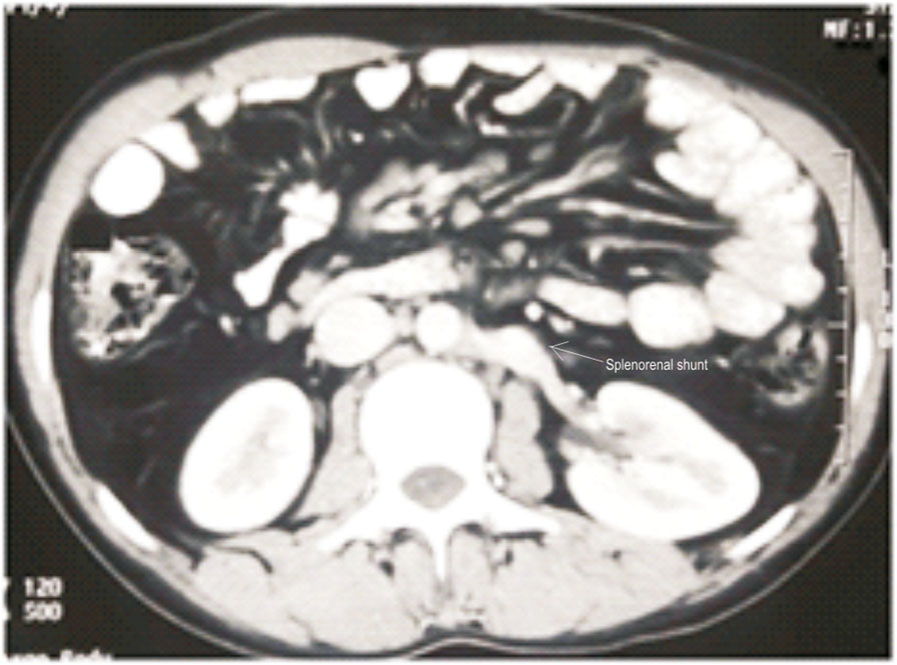
Cross section image showing absent spleen with patent end to side splenorenal shunt

**Fig. 7:**
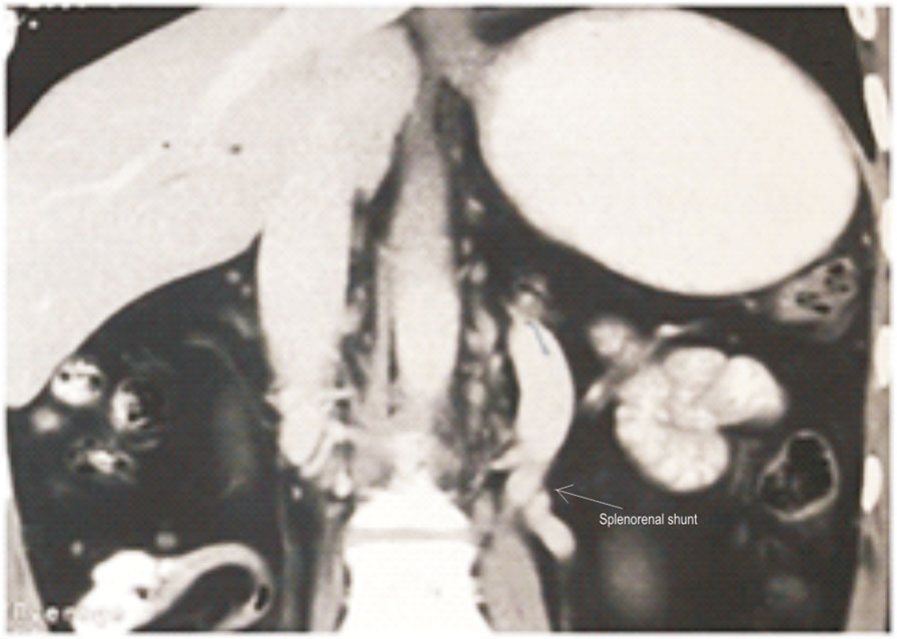
Coronal section image showing absent spleen with patent end to side splenorenal shunt

**Fig. 8:**
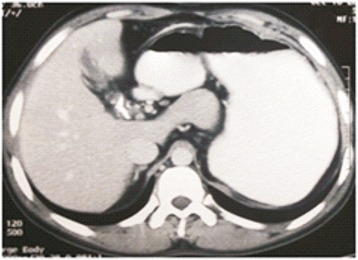
Fewer collaterals around gallbladder and hepatoduodenal ligament with decreased dilatation of intrahepatic biliary radicals

At 4 months follow-up, UGIE (Fig. 5) showed no esophageal varices, no ulcers with normal stomach. The contrast enhanced computed tomography confirmed patent shunt (Figs. 6 and 7), normal liver, and regression of periportal, perigallbladder collaterals and proximal biliary dilatation with normal kidneys (Fig. 8). Hemogram, liver function tests, renal function tests and urine examination were normal.

The three year follow-up in July 2016 was without any recurrence of variceal bleed, evidence of hypersplenism, encephalopathy and ascites. The shunt was patent on Duplex ultrasound of the abdomen with normal hemogram (hemoglobin- 10.2 g/dl, total leucocyte count 9800/cumm and platelets 2.5 lakh/cumm), normal liver function tests, renal function tests and urine examination.

## Discussion

Present case provides the first evidence that retro-aortic LRV can withstand the extra volume of portal blood flow through the proximal shunt without causing renal venous hypertension and thus providing the effective portal decompression so as to treat all the components of EHPVO.

EHPVO is a common cause of portal hypertension and variceal bleeding in children and young adults, in third world countries. The aetiology of EHPVO is largely idiopathic (65%) with omphalitis, umbilical vein cathetorization and intra-abdominal sepsis responsible in some of the cases [7].

Shunt surgery is indicated in a group of patients where variceal bleeding fails to respond to endoscopic management, portal hypertensive gastropathy, symptomatic hypersplenism, portal biliopathy, ectopic varices and in patients who do not have access to endoscopic facilities and expertise [2, 8].

In 1947, Dr. Robert Linton [9] first reported splenectomy with PSRS. Since then it has gained more popularity in developing countries where EHPVO is a major cause of portal hypertension [2, 10] with associated symptomatic hypersplenism. PSRS in EHPVO is established as the one time procedure that prevents variceal bleeding with rebleeding rate of 0–2%, no mortality and no encephalopathy in the postoperative period, and low incidence of post splenectomy infection [2, 3, 7].

Additionally PSRS also cures EHPVO related problems other than variceal bleed like portal hypertensive gastropathy and portal biliopathy in majority of patients [11]. PSRS is found to be more effective in relieving any associated hypersplenism [10] and it does not need any use of natural or synthetic grafts unlike mesocaval shunt. Post splenectomy infection rates in developing world is low as compared to western countries [2, 12] possibly because children living in poor hygienic conditions develop immunity against serious infections after recurrent attacks of gastrointestinal infections.

In the presence of normal anatomy PSRS is done after splenectomy by anastomosing the end of splenic vein with the side of left renal vein taking advantage of its long length as it crosses over the aorta before joining IVC. Left renal vein usually lies anterior to the aorta but in 1.8 – 3.4% cases there is a single LRV in retro-aortic position before entering IVC [4]. This position restricts its mobility, reduces the advantages of its greater length [5] and its compression by aorta predisposes for renal venous hypertension [6]. These abnormalities mandate that attention should be given to some important aspects leading to a successful PSRS with retro-aortic LRV. These include dissection of greater length of splenic vein from the pancreas, complete mobilization of the LRV from the retroperitoneum extending from left margin of aorta to renal hilum, ligation and division of left adrenal and gonadal veins to achieve greater mobility of LRV and measurement of pressure gradients across anastomosis and between LRV and IVC to ensure effective shunt, as were done in present case.

The renal venous hypertension is diagnosed when a pressure gradient of ≥10 mmHg exists between LRV and IVC and there is no pressure gradient across the anastomosis [13]. Though there is one report of two cases stating that retroaortic renal vein does not preclude successful formation of a Warren shunt in cirrhotic patients [14]. In these patients it is only selective portal decompression in comparison to PSRS which is a total shunt with total portal venous blood diverted through the splenorenal shunt into IVC through retroaortic left renal vein. This may result in the renal venous hypertension eventually affecting the complete portal decompression.

End to side PSRS with retro-aortic LRV in portal cavernoma may fail for anatomical reasons or due to incomplete portal decompression from the extra volume of flow through the shunt unlike distal shunt where portal decompression is selective. Complete portal decompression is essential to treat all the manifestations of extrahepatic portal hypertension like variceal bleeding, gastropathy and biliopathy. The present case shows that PSRS with retroaortic LRV is feasible, safe and effective for the treatment of all the components of EHPVO related portal hypertension as variceal bleed, gastropathy, biliopathy and symptomatic hypersplenism.

There are many non surgical options available for the treatment of portal hypertension. There is limited experience with the medical treatment by use of beta blockers in EHPVO [15]. Endoscopic management (sclerotherapy / variceal ligation) of esophageal varices is found to be easy and effective in controlling acute esophageal variceal bleeding in 90–95% patients [8]. But these endoscopic methods are associated with 16% risk of esophageal variceal recurrence, 11% new varices in stomach [2], more risk of portal biliopathy [16] and 5% mortality from bleeding [2] as the portal system is not decompressed.

Transjugular intrahepatic portosystemic shunt (TIPS), though is a good short term solution for failed endotherapy or for prevention of recurrent bleed in patients with end stage liver disease who are candidates for liver transplant, its use in EHPVO is limited. Percutaneous transhepatic occlusion of coronary vein with obliteration of bleeding collateral esophageal varices is another effective modality but for this again patency of PV is a prerequisite [10]. Symptomatic acute PVT in contrast to EHPVO can be treated by removal of thrombus through the transjugular route [8].

Hypersplenism can also be managed by partial splenic embolization using steel coils or gel foam causing infarction of 60–70% of splenic substance [8]. However, this may be difficult to achieve without complications in large spleens associated with EHPVO patients.

There are many other operations including devascularisation and different types of shunts which were performed for the treatment of EHPVO. Devascularisation has been associated with rebleeding rates of 30–40% as the portal system was not decompressed [2].

The main advantage of Warren (selective) shunt with lower incidence of encephalopathy than the total shunts is not relevant in EHPVO as post shunt encephalopathy does not occur in them [2]. Also it will not be useful for the treatment of portal biliopathy, ectopic varices and it may not reverse large spleen related discomfort and hypersplenism after splenic vein decompression [17].

Side to side splenorenal shunt has been shown to have excellent long term patency rate with low rate of recurrent bleeding. It also reduces the size of spleen and cures hypersplenism [7]. Nevertheless, we thought it may not be technically feasible to bring both splenic and left renal vein together for anastomosis due to the retroaortic course of LRV along with massive splenomegaly.

Mesoportal shunt (Rex shunt) was proposed as the best option for EHPVO patients and patent left portal vein as it maintains portal flow through liver. It is associated with disadvantages like requirement of graft, more lengthy procedure, does not provide treatment for symptomatic splenomegaly and its limited experience in adults [18].

## Conclusion

PSRS is a feasible, safe and effective procedure when done with retro-aortic LRV for the treatment of portal hypertension related to EHPVO on the condition that attention is paid to key technical considerations including pressure studies to ensure effective shunt.

## Abbreviations

EHPVO: Extraheaptic portal vein obstruction
EVL: Endoscopic variceal ligation
IVC: Inferior vena cava
LRV: Left renal vein
PSRS: Proximal splenorenal shunt
UGIE: Upper gastrointestinal endoscopy
